# Concordance of Cephalometric Classifications of Divergence in Orthodontics

**DOI:** 10.7759/cureus.94243

**Published:** 2025-10-09

**Authors:** Maria E Saadeh

**Affiliations:** 1 Orthodontics, Lebanese University Faculty of Dental Medicine, Beirut, LBN

**Keywords:** cephalometric analysis, facial divergence classification, measures of agreement, orthodontic diagnosis, skeletal vertical pattern

## Abstract

Background

Vertical skeletal pattern is central to orthodontic diagnosis. We compared five cephalometric methods-Steiner (Sella-Nasion to mandibular plane, SN/GoGn), Downs (mandibular plane to Sella-Nasion, MP/SN), Tweed (Frankfort-mandibular plane angle, FMA), Arnett (palatal plane to mandibular plane, PP/MP), and Ricketts (facial axis angle, FAA)-for classifying facial divergence.

Methods

We retrospectively analyzed 300 lateral cephalograms (114 males, 186 females; mean age 26.4 ± 9.9 years) from skeletal Class I adults (A point-Nasion-B point angle, ANB, 0° < ANB < 4°). Each method classified subjects as hypo-, normo-, or hyperdivergent. Agreement was assessed using Cohen’s kappa (κ), and ordinal association using Kendall’s tau-b (τ_b).

Results

Divergence prevalence varied by method: hypodivergent 16% (48/300) with MP/SN to 30% (90/300) with SN/GoGn; hyperdivergent 15.3% (46/300) with PP/MP to 25% (75/300) with MP/SN. Agreement was substantial for SN/GoGn vs MP/SN (κ = 0.656, p < 0.001), moderate for several other pairs (κ ≈ 0.49-0.54), and lowest when FAA was compared (κ ≈ 0.11-0.22). Overall agreement was fair (κ = 0.375, p < 0.001).

Conclusions

Cephalometric methods are not interchangeable for vertical classification. Reporting at least two complementary indices is advisable; FAA should not be used alone. Standardized thresholds and multi-index confirmation may improve diagnostic consistency.

## Introduction

Vertical facial divergence is a crucial aspect of craniofacial morphology and plays a significant role in orthodontic diagnosis, treatment planning, and long-term stability. This dimension is commonly assessed by relating the mandibular plane to cranial or palatal reference planes, classifying patients as hypo-, normo-, or hyperdivergent.

Various cephalometric analyses have been developed to classify facial divergence, each using different landmarks and reference planes: Steiner’s Mandibular Plane Angle (MPA) [1], Downs’ Mandibular Plane to SN angle (MP/SN) [2], Tweed’s Mandibular and Frankfort horizontal planes (Go-Me/Po-Or) [3], Arnett’s Palatal Plane to Mandibular Plane angle (PP/MP) [4], and Ricketts’ Facial Axis Angle (FAA) [5].

Previous studies have reported varying levels of agreement between these analyses, ranging from slight to moderate: Aguilar-Perez FJ et al. [6] found a fair agreement between the Björk-Jarabak and Ricketts analyses, and Herreros del Pozo A et al. [7] reported a similar level of agreement between the Steiner and Ricketts analyses. However, Claro CA et al. [8] demonstrated only a slight concordance between the Jarabak and Ricketts analyses, unlike Benedicto EN et al. [9], who showed a fair agreement between the same methods. Plaza SP et al. [10] reported a strong association between sagittal and vertical skeletal patterns across the three sagittal classification systems evaluated: Class II malocclusion showed a higher prevalence of hyperdivergence, whereas Class III showed a higher prevalence of hypodivergence.

Given this inconsistency in the literature, this study aimed to evaluate the agreement between five widely used cephalometric methods in categorizing facial divergence among skeletal Class I adults.

The objectives of this research were to compare the concordance between these methods in classifying individuals as hypodivergent, normodivergent, or hyperdivergent; to identify discrepancies and potential biases arising from variations in reference planes, landmark identification, and sample characteristics; and to highlight the clinical implications of cephalometric classification discrepancies for orthodontic diagnosis, treatment planning, and long-term stability.

## Materials and methods

Study design and population

This cross-sectional study analyzed lateral cephalometric radiographs of 300 adults (114 males and 186 females; mean age, 26.4 ± 9.9 years) selected from the Lebanese University Faculty of Dental Medicine database.

The inclusion criteria were defined as females aged ≥ 16 years and males aged ≥ 18 years, skeletal Class I (0° < ANB < 4°), Class I canine and molar relationships, and full permanent dentition.

Excluded were individuals with systemic disease, craniofacial anomalies, a history of orthodontic and/or surgical treatment involving the head and neck, missing posterior teeth (except third molars), or prostheses that could alter the vertical dimension.

Anonymized retrospective data were used, minimizing any potential risk to patient privacy. Ethical approval was obtained from the Institutional Review Board at the Lebanese University (CUEMB 35/AA).

Radiographic analysis

**Figure 1:**
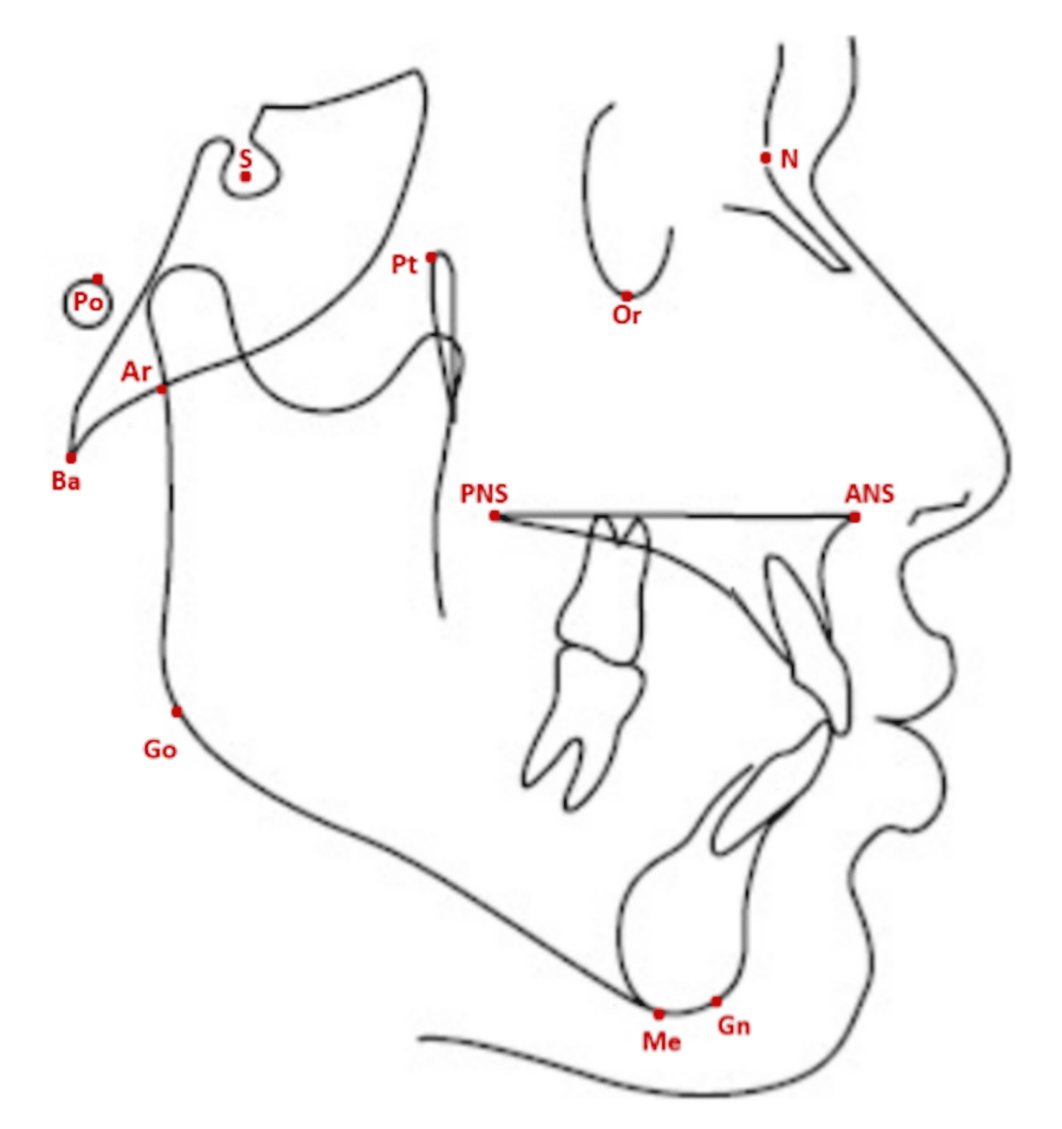
Cephalometric hard tissue landmarks used in the study. N: Nasion; S: Sella; Po: Porion; Pt: Pterygoid; Or: Orbitale; Ar: Articulare; Ba: Basion; ANS: Anterior Nasal Spine; PNS: Posterior Nasal Spine; Go: Gonion; Me: Menton; Gn: Gnathion. Original illustration created by the author.

**Figure 2:**
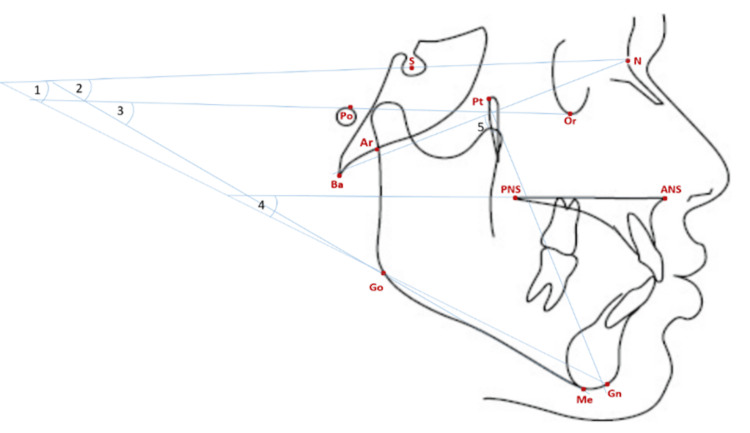
Angular parameters of facial divergence. 1: Steiner; 2: Downs; 3: Tweed; 4: Arnett; 5: Ricketts. Original illustration created by the author.

All lateral cephalograms were acquired in the natural head position using the same unit (Kodak 8000C, Carestream Health, Rochester, NY, USA). One examiner digitized the images in Viewbox 4.0.1.6 using standard landmark definitions (Figure 1) to generate angular cephalometric measurements (Figure 2). Facial divergence was then classified according to established criteria for each analysis, as shown in Table 1.

**Table 1:** Cephalometric classification of facial divergence.

Analysis	Measurement	Interpretation	Classification
Steiner CC [1]	Mandibular Plane Angle (MPA)	Angle formed by the Sella-Nasion (SN) plane and the mandibular plane (Go-Gn)	Hypodivergent < 28° Normodivergent 28-36° Hyperdivergent > 36°
Downs WB [2]	MP/SN angle	Angle formed by the Sella-Nasion (SN) plane and the mandibular plane (Go-Me)	Hypodivergent < 27° Normodivergent 27-37° Hyperdivergent > 37°
Tweed CH [3]	FMA	Angle between the mandibular plane (Go-Me) and the Frankfort horizontal plane (Po-Or)	Hypodivergent < 20° Normodivergent 20-28° Hyperdivergent > 28°
Arnett GW et al. [4]	PP/MP angle	Angle formed by the palatal plane (ANS-PNS) and the mandibular plane (Go-Gn)	Hypodivergent < 20° Normodivergent 20-30° Hyperdivergent > 30°
Ricketts RM et al. [5]	Facial Axis Angle (FAA)	Angle formed by the facial axis line (Ba-N) and the Pterygoid-Gnathion line (Pt-Gn)	Hypodivergent > 93° Normodivergent 87-93° Hyperdivergent < 87°

Reliability

To assess intra-examiner reproducibility, the same examiner re-digitized 30 randomly selected radiographs after three weeks. For inter-examiner reproducibility, a second examiner independently digitized another set of 30 blinded radiographs.

Statistical analysis

An a priori power analysis (80%, effect size = 0.50, α = 0.05) indicated a minimum sample size of n ≥ 128; the final n = 300 exceeded this requirement. Agreement between methods for the three-level divergence classification (hypo-/normo-/hyperdivergent) was quantified using Cohen’s kappa (κ) and interpreted following Landis & Koch benchmarks (slight < 0.20; fair 0.21-0.40; moderate 0.41-0.60; substantial 0.61-0.80; almost perfect 0.81-1.00) [11]. Kendall’s tau-b (τ_b) assessed ordinal associations across the ordered categories. Linear associations among continuous angles were evaluated using the Pearson correlation coefficient (r).

For reliability, intra- and inter-examiner reproducibility of continuous angles (SN/GoGn, MP/SN, FMA, PP/MP, FAA) were evaluated using ICC(2,1) (two-way random effects, absolute agreement, single measures). For the three-level divergence categories, weighted Cohen’s κ was calculated.

Descriptive statistics (mean ± SD, range) were reported for age and all angular measurements, and cross-tabulations of classifications by method were provided.

As a sensitivity analysis, agreement (weighted κ) and ordinal association (τ_b) were re-evaluated after stratifying by age (<21, 21-29, 30-39, ≥ 40 years).

Statistical significance was set at α = 0.05. All analyses were performed using SPSS® v24.0 (IBM®) and Stata/SE™ 11.1.

## Results

Descriptive statistics

The sample included n = 300 adults (mean age: 26.41 ± 9.93 years; range: 16-61.58). Summary measures for all angles are presented in Table 2.

**Table 2:** Descriptive statistics of the total sample. SN/GoGn: Sella-Nasion to mandibular plane; MP/SN: Mandibular plane to Sella-Nasion; FMA: Frankfort-mandibular plane angle; PP/MP: Palatal plane to mandibular plane; FAA: Facial axis angle. Age is expressed in years; all angles are expressed in degrees (°).

Measurement	Minimum	Maximum	Mean	SD
Age (years)	16	61.58	26.41	9.93
SN/GoGn (Steiner)	16.3°	47.8°	31.36°	5.74
MP/SN (Downs)	17.9°	49.3°	33.38°	5.83
FMA (Tweed)	8.1°	40.9°	24.16°	4.94
PP/MP (Arnett)	8.1°	39.9°	24.38°	5.68
FAA (Ricketts)	77.8°	102.0°	89.90°	4.06

Reliability

Intra-examiner reproducibility for the angular measurements was excellent across all variables (ICC(2,1) range: 0.94-0.99; 95% CI: 0.91-1.00). Inter-examiner reproducibility was likewise excellent (ICC(2,1) range: 0.92-0.98; 95% CI: 0.88-0.99). For the three-category divergence classification (hypo-/normo-/hyperdivergent), test-retest agreement was substantial to almost perfect (weighted κ: intra = 0.99, inter = 0.98), indicating that both landmark digitization and derived classifications were stable and reproducible.

Prevalence of divergence patterns by method

The prevalence varied by method: hypodivergent prevalence ranged from 16% (MP/SN) to 30% (SN/GoGn); normodivergent from 51.7% (SN/GoGn) to 60.7% (PP/MP); and hyperdivergent from 15.3% (PP/MP) to 25% (MP/SN) (Table 3).

**Table 3:** Distribution of facial divergence patterns according to different cephalometric measurements. SN/GoGn: Sella-Nasion to mandibular plane; MP/SN: Mandibular plane to Sella-Nasion; FMA: Frankfort-mandibular plane angle; PP/MP: Palatal plane to mandibular plane; FAA: Facial axis angle.

Group	SN/GoGn n (%)	MP/SN n (%)	FMA n (%)	PP/MP n (%)	FAA n (%)
Overall (n = 300)					
Hypodivergent	90 (30.0)	48 (16.0)	63 (21.0)	72 (24.0)	67 (22.3)
Normodivergent	155 (51.7)	177 (59.0)	180 (60.0)	182 (60.7)	166 (55.3)
Hyperdivergent	55 (18.3)	75 (25.0)	57 (19.0)	46 (15.3)	67 (22.3)
Females (n = 186)					
Hypodivergent	50 (26.9)	24 (12.9)	37 (19.9)	48 (25.8)	43 (23.1)
Normodivergent	106 (57.0)	119 (64.0)	111 (59.7)	115 (61.8)	97 (52.2)
Hyperdivergent	30 (16.1)	43 (23.1)	38 (20.4)	23 (12.4)	46 (24.7)
Males (n = 114)					
Hypodivergent	40 (35.1)	24 (21.1)	26 (22.8)	24 (21.1)	24 (21.1)
Normodivergent	49 (43.0)	58 (50.9)	69 (60.5)	67 (58.8)	69 (60.5)
Hyperdivergent	25 (21.9)	32 (28.1)	19 (16.7)	23 (20.2)	21 (18.4)

Sex-stratified distributions

Patterns in females and males mirrored the overall sample. In females, hypodivergence had the highest prevalence with SN/GoGn (26.9%) and the lowest with MP/SN (12.9%); normodivergence was most prevalent with MP/SN (64%) and least with FAA (52.2%); hyperdivergence was most prevalent with FAA (24.7%) and least with PP/MP (12.4%).

In males, hypodivergence was most prevalent with SN/GoGn (35.1%) and least with MP/SN, PP/MP, and FAA (21.1%); normodivergence was most prevalent with FMA/FAA (60.5%) and least with SN/GoGn (43%); hyperdivergence was most prevalent with MP/SN (28.1%) and least with FMA (16.7%) (Table 3).

Concordance by prevalence

Concordance by category was highest between SN/GoGn and MP/SN. All 55/55 subjects classified as hyperdivergent by SN/GoGn were likewise hyperdivergent by MP/SN; among SN/GoGn-normodivergent individuals (n = 155), 135/155 were MP/SN-normodivergent; and 55/90 SN/GoGn-hypodivergent were also hypodivergent by both FMA and PP/MP. The same trend held by sex, with the only difference observed in males within hypodivergence, where MP/SN showed the highest concordance (Table 4).

**Table 4:** Distribution of facial divergence patterns according to SN/GoGn. SN/GoGn: Sella-Nasion to mandibular plane; MP/SN: Mandibular plane to Sella-Nasion; FMA: Frankfort-mandibular plane angle; PP/MP: Palatal plane to mandibular plane; FAA: Facial axis angle.

Group	MP/SN	FMA	PP/MP	FAA
Overall (n = 300)				
Hypodivergent (n = 90)	48	55	55	36
Normodivergent (n = 155)	135	127	125	92
Hyperdivergent (n = 55)	55	37	31	21
Females (n = 186)				
Hypodivergent (n = 50)	24	33	34	22
Normodivergent (n = 106)	93	86	85	60
Hyperdivergent (n = 30)	30	22	15	14
Males (n = 114)				
Hypodivergent (n = 40)	24	22	21	14
Normodivergent (n = 49)	42	41	40	32
Hyperdivergent (n = 25)	25	15	16	7

All 48/48 MP/SN-hypodivergent cases were SN/GoGn-hypodivergent. Among MP/SN-normodivergent (n = 177), 137/177 were FMA-normodivergent (highest concordance for this stratum). Of MP/SN-hyperdivergent (n = 75), 55/75 were SN/GoGn-hyperdivergent. By sex, the only difference was in females within normodivergence, where SN/GoGn showed the highest concordance (Table 5).

**Table 5:** Distribution of facial divergence patterns according to MP/SN. SN/GoGn: Sella-Nasion to mandibular plane; MP/SN: Mandibular plane to Sella-Nasion; FMA: Frankfort-mandibular plane angle; PP/MP: Palatal plane to mandibular plane; FAA: Facial axis angle.

Group	SN/GoGn	FMA	PP/MP	FAA
Overall (n = 300)				
Hypodivergent (n = 48)	48	36	34	17
Normodivergent (n = 177)	135	137	128	98
Hyperdivergent (n = 75)	55	44	35	28
Females (n = 186)				
Hypodivergent (n = 24)	24	18	18	8
Normodivergent (n = 119)	93	90	82	61
Hyperdivergent (n = 43)	30	28	16	19
Males (n = 114)				
Hypodivergent (n = 24)	24	18	9	9
Normodivergent (n = 58)	42	47	46	37
Hyperdivergent (n = 32)	25	16	19	9

Among FMA-hypodivergent (n = 63), 55/63 were SN/GoGn-hypodivergent. Of FMA-normodivergent (n = 180), 141/180 were PP/MP-normodivergent. Among FMA-hyperdivergent (n = 57), 44/57 were MP/SN-hyperdivergent. In females, MP/SN provided the highest concordance within normodivergence (Table 6).

**Table 6:** Distribution of facial divergence patterns according to FMA. SN/GoGn: Sella-Nasion to mandibular plane; MP/SN: Mandibular plane to Sella-Nasion; FMA: Frankfort-mandibular plane angle; PP/MP: Palatal plane to mandibular plane; FAA: Facial axis angle.

Group	SN/GoGn	MP/SN	PP/MP	FAA
Overall (n = 300)				
Hypodivergent (n = 63)	55	36	50	30
Normodivergent (n = 180)	127	137	141	111
Hyperdivergent (n = 57)	37	44	29	24
Females (n = 186)				
Hypodivergent (n = 37)	33	18	32	20
Normodivergent (n = 111)	86	90	89	67
Hyperdivergent (n = 38)	22	28	17	18
Males (n = 114)				
Hypodivergent (n = 26)	22	18	18	10
Normodivergent (n = 69)	41	47	52	44
Hyperdivergent (n = 19)	15	16	12	6

Among PP/MP-hypodivergent (n = 72), 55/72 were SN/GoGn-hypodivergent. Of PP/MP-normodivergent (n = 182), 141/182 were FMA-normodivergent. Among PP/MP-hyperdivergent (n = 46), 35/46 were MP/SN-hyperdivergent. In females, FMA showed the highest concordance within hyperdivergence (Table 7).

**Table 7:** Distribution of facial divergence patterns according to PP/MP. SN/GoGn: Sella-Nasion to mandibular plane; MP/SN: Mandibular plane to Sella-Nasion; FMA: Frankfort-mandibular plane angle; PP/MP: Palatal plane to mandibular plane; FAA: Facial axis angle.

Group	SN/GoGn	MP/SN	FMA	FAA
Overall (n = 300)				
Hypodivergent (n = 72)	55	34	50	33
Normodivergent (n = 182)	125	128	141	105
Hyperdivergent (n = 46)	31	35	29	15
Females (n = 186)				
Hypodivergent (n = 48)	34	18	32	22
Normodivergent (n = 115)	85	82	89	64
Hyperdivergent (n = 23)	15	16	17	11
Males (n = 114)				
Hypodivergent (n = 24)	21	16	18	11
Normodivergent (n = 67)	40	46	52	41
Hyperdivergent (n = 23)	16	19	12	4

Among FAA-hypodivergent (n = 67), 36/67 were SN/GoGn-hypodivergent. Of FAA-normodivergent (n = 166), 111/166 were FMA-normodivergent. Among FAA-hyperdivergent (n = 67), 28/67 were MP/SN-hyperdivergent. No sex differences in concordance were observed for FAA-based strata (Table 8).

**Table 8:** Distribution of facial divergence patterns according to the facial axis angle. SN/GoGn: Sella-Nasion to mandibular plane; MP/SN: Mandibular plane to Sella-Nasion; FMA: Frankfort-mandibular plane angle; PP/MP: Palatal plane to mandibular plane; FAA: Facial axis angle.

Group	SN/GoGn	MP/SN	FMA	PP/MP
Overall (n = 300)				
Hypodivergent (n = 67)	36	17	30	33
Normodivergent (n = 166)	92	98	111	105
Hyperdivergent (n = 67)	21	28	24	15
Females (n = 186)				
Hypodivergent (n = 43)	22	8	20	22
Normodivergent (n = 97)	60	61	67	64
Hyperdivergent (n = 46)	14	19	18	11
Males (n = 114)				
Hypodivergent (n = 24)	14	9	10	11
Normodivergent (n = 69)	32	37	44	41
Hyperdivergent (n = 21)	7	9	6	4

Agreement and ordinal association

Age-stratified analyses yielded the same qualitative conclusions; for parsimony, only unstratified results are reported.

Pairwise Cohen’s kappa (κ) indicated substantial agreement between Steiner (SN/GoGn) and Downs (MP/SN) (κ = 0.656, p < 0.001), and moderate agreement for Steiner × Tweed (FMA) (κ = 0.544), Tweed × Arnett (PP/MP) (κ = 0.521), Downs × Tweed (κ = 0.510), and Steiner × Arnett (κ = 0.494) (all p < 0.001). The lowest κ was observed for Downs × Ricketts (FAA) (κ = 0.110, p = 0.016).

Kendall’s tau-b (τ_b) values were small overall, ranging from 0.000-0.207, with the largest for Steiner × Downs (τ_b = 0.207, p < 0.001); some pairs (e.g., Steiner × Arnett) even showed non-significant τ_b despite significant κ (Table 9).

**Table 9:** Strength of agreement for cephalometric measurements. *Statistically significant at p < 0.05; **Statistically significant at p < 0.01. Kappa = Cohen’s kappa (κ); Kendall’s tau = Kendall’s tau-b (τ_b).

Matches	Kappa (κ)	p-value (Kappa)	Kendall’s tau (τᵦ)	p-value (Kendall)
Steiner × Downs	0.656	<0.001**	0.207	<0.001**
Steiner × Tweed	0.544	<0.001**	0.035	0.001**
Steiner × Arnett	0.494	<0.001**	0.002	0.458
Steiner × Ricketts	0.17	<0.001**	0.019	0.018*
Downs × Tweed	0.51	<0.001**	0.044	<0.001**
Downs × Arnett	0.393	<0.001**	0.091	<0.001**
Downs × Ricketts	0.11	0.016*	0.013	0.046*
Tweed × Arnett	0.521	<0.001**	0.017	0.025*
Tweed × Ricketts	0.222	<0.001**	0	0.796
Arnett × Ricketts	0.15	<0.001**	0.01	0.083

Overall and group-specific agreement

Across all methods, the overall agreement scored κ = 0.375 (p < 0.001). Agreement by category was moderate for the hypodivergent (κ = 0.456) and hyperdivergent (κ = 0.415) groups, and fair for the normodivergent group (κ = 0.290). The overall ordinal association was τ_b = 0.034 (p < 0.001) (Table 10).

**Table 10:** Strength of agreement in each group. **Statistically significant at p < 0.01. Kappa: Cohen’s kappa (κ); Kendall’s tau: Kendall’s tau-b (τ_b).

Group	Kappa (κ)	p-value (Kappa)	Kendall’s tau (τᵦ)	p-value (Kendall)
Overall	0.375	<0.001**	0.034	<0.001**
Hypodivergent	0.456	<0.001**	-	-
Normodivergent	0.29	<0.001**	-	-
Hyperdivergent	0.415	<0.001**	-	-

Correlation among angular measures

A strong, statistically significant correlation was observed between SN/GoGn and MP/SN (r = 0.996), MP/SN and FMA (r = 0.866), and FMA and PP/MP (r = 0.827). By contrast, FAA showed weaker and often negative correlations with these angles (e.g., SN/GoGn × FAA: r = -0.377; MP/SN × FAA: r = -0.369; FMA × FAA: r = -0.421) (Table 11).

**Table 11:** Correlation coefficients among various vertical skeletal measurements. Pearson correlation coefficients (r). **Statistically significant at p < 0.01. SN/GoGn: Sella-Nasion to Gonion-Gnathion angle (Steiner’s Mandibular Plane Angle); MP/SN: Mandibular Plane to Sella-Nasion angle (Downs’ Mandibular Plane Angle); FMA: Frankfort-Mandibular Plane Angle (Tweed’s FMA); PP/MP: Palatal Plane to Mandibular Plane angle (Arnett’s Analysis); FAA: Facial Axis Angle (Ricketts’ Analysis).

	MP/SN	FMA	PP/MP	FAA
SN/GoGn	0.996**	0.861**	0.799**	-0.377**
MP/SN		0.866**	0.804**	-0.369**
FMA			0.827**	-0.421**
PP/MP				-0.396**

## Discussion

Orthodontists routinely assess the vertical skeletal pattern; however, different reference planes can classify the same patient differently. In addition, the scarcity of comparative studies in the literature makes it challenging for clinicians to select the most appropriate analysis for accurate judgment.

The present study aimed to evaluate the strength of agreement among five commonly used methods for assessing facial divergence in skeletal Class I individuals. To our knowledge, this is the first study to include these five vertical pattern assessment angles in a large sample of Class I individuals.

The main finding of this study was that the choice of analysis affected both the prevalence of divergence categories and the degree of concordance between indices. Agreement was substantial between SN/GoGn and MP/SN (κ = 0.656, p < 0.001), moderate for several other pairs, and lowest when FAA was involved. Overall agreement across all methods was fair. Correlations were strongest among SN/GoGn, MP/SN, and FMA, whereas FAA correlated weakly, and often negatively, with these measures.

Hypodivergence was most frequently recorded using Steiner’s SN/GoGn, normodivergence with Arnett’s PP/MP, and hyperdivergence with Downs’ MP/SN. The findings differ from those of Mourgues T et al. [12], where Steiner’s values tended more toward hyperdivergence compared with Ricketts, Björk-Jarabak, and McNamara. This discrepancy is likely due to methodological differences between the studies. In fact, there is variation in the definition of the MP among the above-mentioned authors. Steiner CC [1] and Arnett GW [4] both use Gonion and Gnathion to draw the MP, whereas Downs [2] defines MP as the line passing through Gonion and Menton. The Go-Me line reflects mandibular body inclination and uses an extreme inferior point [13], while Go-Gn provides a measure of mandibular rotation and chin position [14].

Our study found the strongest agreement (Cohen’s Kappa) and the highest correlation coefficient between Downs’ and Steiner’s angular measurements. Both measurements include the anterior cranial base length (SN) and the MP in their definition, differing only in the landmarks defining the MP. Although Menton may be easier to identify consistently than Gnathion, Go-Gn is considered more stable and less affected by vertical growth than Go-Me [15,16]. Our findings align with those of Ahmed M et al. [16] and Türker G et al. [17-18], who reported robust associations for these indices.

The weakest correlation coefficients were found between Ricketts’ FAA and all other measurements, which also showed the lowest agreement scores. This may be explained by the fact that the facial axis angle diverges from other vertical pattern measurements, as it is not solely an angular indicator of the vertical dimension between the jaws. It also reflects sagittal direction, overall growth direction, and cranial base inclination, which is directly influenced by cranial base morphology. In a recent study [18], Ricketts’ FAA was found useful for growth pattern evaluation, though not always in agreement or strongly correlated with other vertical skeletal measurements. In our sample, composed entirely of adult patients where growth is no longer a variable, the weak correlation observed can thus be interpreted as a true disagreement between Ricketts’ and other analyses.

Steiner’s vertical pattern angle (SN/GoGn) was also found to be moderately correlated (r = 0.86) and in agreement (κ = 0.544) with Tweed’s FMA angle in the present study. Our results concur with previous research [13,14,19], indicating that SN/GoGn is systematically larger than FMA by approximately 6°-7°, reflecting the average angular relationship between FH and SN. Similarly, Roy P et al. [15] and Ahmed M et al. [16] reported that these two measurements are reliable indicators for vertical pattern assessment.

Arnett’s PP/MP angle directly relates the jaws and is less influenced by the cranial base. It is believed to be among the most reliable planes [20,21], maintaining a stable angular relationship with the cranial base throughout life [21,22]. In the present study, its strongest correlation and agreement were with Tweed’s FMA angle. Several studies have shown that the palatal plane is roughly parallel to the Frankfort horizontal [13,14], yet it is easier to identify.

When genders were analyzed separately, the classifications largely mirrored those of the overall sample. Given that this study included more females (n = 186) than males (n = 114), and since no prior study with balanced gender distribution exists for comparison, this observation warrants confirmation in future research with equal male-female representation.

Limitations of the study 

Using two-dimensional lateral cephalograms could be considered a limitation; nevertheless, cephalometric measurements remain relevant and reliable [23,24] and expose patients to lower radiation than cone-beam computed tomography (CBCT). Moreover, despite the close interrelationship between sagittal and vertical dimensions, especially considering the various scenarios of mandibular rotation [13], the selection of skeletal Class I individuals was intentional. This approach minimized the confounding influence of sagittal discrepancies on vertical analysis and reduced interpretive variability.

Residual age-related variation cannot be entirely excluded; however, age-stratified sensitivity analyses did not alter the study’s conclusions.

Clinical implications

Because these indices are not interchangeable, relying on a single method may lead to misclassification, particularly near category boundaries. Pairing SN/GoGn with MP/SN or FMA provides a more stable categorization, while FAA should be used cautiously as a standalone diagnostic measure. Recording two complementary indices and reconciling discordant results may enhance diagnostic accuracy, facilitate appropriate biomechanical planning, and improve long-term treatment stability.

## Conclusions

Cephalometric methods for assessing vertical divergence do not fully concord, with agreement ranging from fair overall to substantial between SN/GoGn and MP/SN. Because the choice of method affects classification, clinicians should report at least two complementary indices, for example, SN/GoGn with MP/SN or FMA, and avoid relying solely on FAA for divergence categorization. Adopting standardized thresholds or a multi-index confirmation approach may enhance diagnostic consistency and support more reliable treatment planning.
